# Simulated photovoltaic performance of N719 ruthenium dye sensitised solar cell with a power conversion efficiency exceeding 26% based on electron transport double layer

**DOI:** 10.1039/d5ra06604b

**Published:** 2025-11-14

**Authors:** George G. Njema, Abderrahmane Elmelouky, Edson L. Meyer, Joshua K. Kibet

**Affiliations:** a Department of Chemistry, Egerton University P.O Box 536 – 20115 Egerton Kenya jkibet@egerton.ac.ke; b Laboratory Physics of Condensed Matter (LPMC), University of Chouaib Doukkali El-Jadida Morocco; c University of Fort Hare, Institute of Technology Alice 5700 South Africa

## Abstract

The advancement of modern technologies demands a balanced approach that addresses both environmental sustainability and economic viability. Accordingly, a transition towards renewable energy has become necessary. Among renewable energy options, solar energy is particularly appealing due to its widespread availability and ecological advantages. Dye-sensitized solar cells (DSSCs) offer an environmentally friendly and cost-effective alternative renewable energy. Their benefits include simple fabrication techniques, low-cost materials, and strong performance under low or diffuse light conditions. Herein, we investigate a promising solar cell device of the configuration, FTO/TiO_2_/PC61BM/N719/Spiro-OMeTAD/C, using SCAPS-1D device simulator. The model cell yielded remarkable photovoltaic parameters; power conversion efficiency (PCE) of 26.73%, open-circuit voltage (*V*_oc_) of 1.2284 V, short-circuit current density (*J*_sc_) of 25.11 mA cm^−2^, and a fill factor (FF) of 86.66%. The relative permittivity of the materials used in heterojunction solar cells is often overlooked during the design of new material combinations. This work challenges this approach by studying the impact of permittivity on the physics and performance of solar cells through numerical simulations supported by analytical relationships. The results show that, depending on the cell configuration and material properties, the relative permittivity can have a significant effect on conversion efficiency, though its influence may sometimes be ignored without notable consequences. This work demonstrates that high permittivity materials should be prioritized as partners in the heterojunction with the absorber layer. The present configuration makes solar cells more robust against non-idealities, which are likely to occur in the early stages of development before full optimization of the device is realized.

## Abstract 2

The advancement of modern technologies demands a balanced approach that addresses both environmental sustainability and economic viability.

## Graphical abstract

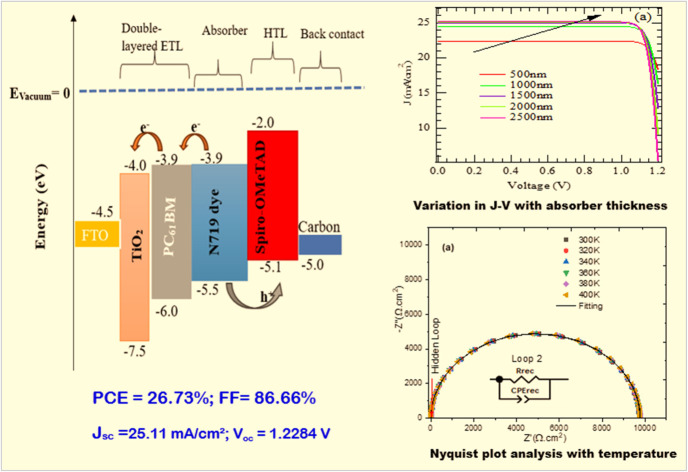

## 1 Introduction

The advancement of modern technologies demands a balanced approach that addresses both environmental sustainability and economic viability. Accordingly, the global transition toward renewable energy has become imperative. Among the various renewable options, solar energy remains particularly attractive due to its abundance, scalability, and minimal environmental impact. In this context, DSSCs have emerged as a promising photovoltaic technology, offering a sustainable and cost-effective pathway to clean energy generation. DSSCs are characterized by simple fabrication procedures, low-cost materials, and strong performance even under low or diffuse light conditions, making them ideal for both indoor and outdoor applications.^1^

This work presents an innovative cell structure with PC61BM and TiO_2_ as electron transport double layers and N719 dye for superior light harvesting, working in tandem with optimized charge collection through TiO_2_ and Spiro-OMeTAD layers. Previous studies have investigated similar components separately, but this work distinguishes itself by integrating them into a cohesive structure that dramatically reduces energy losses while enhancing charge injection. The incorporation of a carbon-based back electrode provides additional benefits in terms of affordability and durability compared to conventional metal contacts. This breakthrough design represents a significant advancement in DSSC technology, demonstrating how robust material selection and structural engineering can lead to ground-breaking material recombination. A central focus of this work is the examination of the influence of relative permittivity (dielectric constant) on the overall performance of heterojunction solar cells. Although often neglected in the design of new material combinations, the dielectric properties of the constituent layers can substantially affect charge transport dynamics, the built-in electric field, and recombination rates. Through numerical analysis supported by theoretical considerations, this study demonstrates that materials with higher permittivity can enhance electric field uniformity and device robustness, particularly under non-ideal or early-stage fabrication conditions. Hence, high-permittivity materials should be prioritized when selecting components to pair with the absorber layer in DSSC architectures.

Double electron transport layers (ETLs) present significant advantages compared to single ETL structures in DSSCs solar cells and related devices.^2^ By stacking two complementary ETL materials, charge extraction can be more efficient, interfacial defects are better passivated, and energy level alignment is optimized, leading to reduced electron–hole recombination.^3^ This layered configuration enhances electron mobility and facilitates smoother charge transport, which collectively contribute to improved device parameters such as higher open-circuit voltage (*V*_oc_), increased short-circuit current density (*J*_sc_), greater fill factor (FF), and elevated power conversion efficiency (PCE). Moreover, the dual-layer structure can improve environmental stability by offering better protection against moisture and thermal degradation, while also allowing for more precise tuning of surface and interface properties.

The TiO_2_ ETL is a wide bandgap semi-conductor (3.2 eV) which is well matched with the LUMO of the N719 dye,^4^ enabling ultrafast electron injection on femtosecond-to-picosecond timescales. Although TiO_2_ has moderate electron mobility (10^−7^ to 10^−6^ cm^2^ V^−1^ s^−1^),^5^ its nanostructured, mesoporous form provides large surface area for dye loading, enhancing light harvesting. TiO_2_ also exhibits strong hole-blocking capability and maintains a significant built-in electric field at the dye interface, reducing recombination losses and ensuring directional electron transport. On the other hand, PC61BM, a fullerene derivative, acts as the secondary ETL with higher carrier mobility^6^ forms a cascade alignment with TiO_2_, promoting directional charge transfer. Further, PC61BM effectively passivates TiO_2_ surface defects, eliminates trap states, and reduces interfacial recombination, leading to improved *V*_oc_ and FF. Its smooth, hydrophobic surface also aids in forming a high-quality interface.

The N719 ruthenium based dye is responsible for high solar capture, with strong coverage across the 300–800 nm wavelength range and exhibits high molar extinction coefficient (>10^5^ cm^−1^)^7^ is slightly above the TiO_2_ conduction band, creating a favorable energy offset for rapid electron injection, while its HOMO (−5.5 eV)^8^ aligns with that of Spiro-OMeTAD for efficient hole transfer. Anchoring carboxylate groups ensure firm chemical bonding to TiO_2_, stabilizing the interfacial electric field and maintaining effective charge separation. Spiro-OMeTAD serves as the hole transport layer, offering high hole mobility (∼10^−3^ cm^2^ V^−1^ s^−1^ when appropriately doped) and a wide bandgap (−5.2 eV)^9^ matches that of N719, facilitating efficient hole injection, while its excellent film-forming properties ensure uniform coverage, minimize interfacial defects, and preserve strong local electric fields. The carbon back contact provides a low-cost, conductive, and chemically stable alternative to platinum and other potentially pricy metals. It efficiently collects holes, supports redox reactions for dye regeneration, and it is compatible with scalable, low-temperature fabrication methods. By integrating TiO_2_ and PC61BM as a double ETL, the device benefits from an energy cascade that directs electrons toward the FTO, reduces recombination, and maintains strong interfacial electric fields for effective charge separation. Together with the high absorption efficiency of N719, the excellent hole transport capability of Spiro-OMeTAD, and the durability of the carbon electrode, this architecture inspires promise for designing high performance dye sensitized solar cells that can be injected into the production workflow for scalability and commercialization.

Based on a critical evaluation of the device architecture and its underlying operational photo-physics, the classification of this structure as a DSSC can be justified when viewed within the framework of the ssDSSC family. Although the conventional liquid electrolyte containing an I^−^/I_3_^−^ or Co(ii)/Co(iii) redox couple, as originally demonstrated by O'Regan and Grätzel,^10,11^ is absent, the fundamental photo = electrochemical principles that define a DSSC remain operative. In this configuration, the N719 ruthenium-based dye continues to play its central role as the light-harvesting sensitizer, absorbing incident photons and injecting electrons into the conduction band of the TiO_2_ layer. The subsequent regeneration of the oxidized dye is achieved not by a liquid redox mediator but by a solid hole-transport material which effectively donates electrons to the oxidized dye and transports holes to the counter electrode.^12^ In this context, the incorporation of PC61BM as an electron-transporting interlayer between TiO_2_ and the dye enhances electron extraction and suppresses recombination in hybrid ssDSSC architectures. Thus, PC61BM introduces an additional heterojunction that improves carrier mobility without altering the dye-sensitized injection pathway. Several studies have demonstrated that such hybrid interlayers can optimize energy alignment and broaden light absorption while maintaining the defining dye-sensitized mechanism.^13^ The TiO_2_ film continues to function as the electron-collecting semiconductor scaffold, while the dye serves as the molecular bridge facilitating charge transfer across the interface. Accordingly, the present device architecture FTO/TiO_2_/PC61BM/N719/Spiro-OMeTAD/C is best understood as a solid-state dye-sensitized heterojunction solar cell, which preserves the key operational steps of a DSSC while incorporating solid organic semiconductors to replace the traditional electrolyte.^14–16^

This work introduces a transformative advancement in DSSC technology through the development of a novel solid-state device architecture that redefines electron transport and interfacial engineering. The core innovation lies in the strategic integration of a dual ETL composed of TiO_2_ and PC61BM, configured within an optimized device structure. Unlike conventional single-ETL systems, this dual-layer design establishes an energy cascade that promotes efficient electron extraction, minimizes interfacial recombination, and enhances charge transport dynamics. Beyond structural optimization, the study pioneers a new perspective on the fundamental physics of DSSCs by emphasizing the critical role of material permittivity in determining overall device stability and efficiency. It challenges traditional design paradigms by revealing that the dielectric properties of heterojunction partners significantly influence charge carrier dynamics, defect tolerance, and long-term reliability. This insight establishes enhanced design principle: coupling absorber layers with high-permittivity materials to mitigate performance losses arising from intrinsic and interfacial defects. Notwithstanding the fact that the present cell structure is theoretically examined, the analytical insights offer promise to fabricate an innovative deice of potentially high operational performance.

## 2 Simulation methodology and cell design

In this study, the simulation of the solar cell device was carried out using SCAPS-1D device simulator.^17^ For the purpose of this work, version 3.3.0 of the software was employed. The simulations were performed under standard testing conditions (STCs) to ensure consistency and comparability of results. These conditions include illumination with the AM 1.5 Global solar spectrum, a standard irradiance level of 100 mW cm^−2^, and a temperature of 300 K. Such parameters are representative of typical terrestrial sunlight and are commonly used in photovoltaic research to evaluate and benchmark device performance. With customizable material parameters, it enables precise optimization of device performance. Researchers widely use SCAPS-1D for its reliability, flexibility, and powerful numerical computation in photovoltaic design. This device simulator is based on three coupled differential equations – Poisson equation (eqn 1) and continuity eqn (2) and (3).^4^1
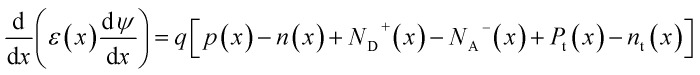
2
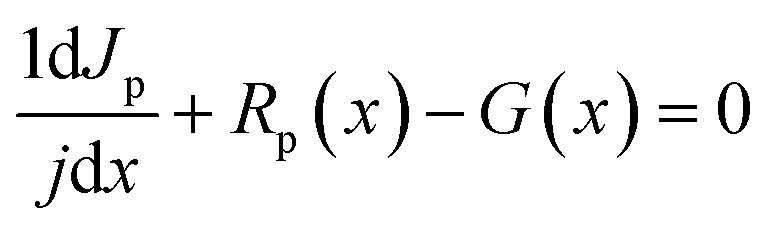
3
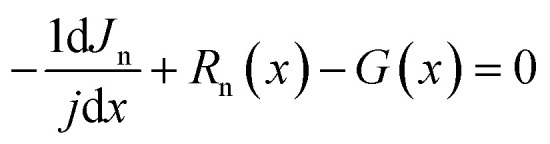
Here, *ε* is the permittivity, *q* is the charge of electron, *ψ* is the electrostatic potential and n is electrons concentration, *p* is free hole concentration, *n*_t_ is trapped electron, *P*_t_ is trapped hole, *N*_D_+ is the ionized donor-like doping and *N*_A_^−^is the ionized acceptor-like doping concentrations, *R*_n_(*x*), *R*_p_(*x*) are electrons and holes recombination rate, *G*(*x*) is the generation rate, *J*_n_ and *J*_p_ are the electron and hole current densities respectively.

**Fig. 1:**
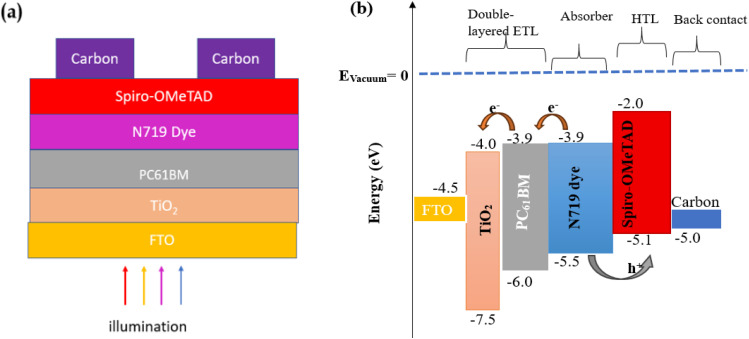
The proposed solar cell's structure (a) and (b) the corresponding energy band diagram.

In SCAPS-1D, Poisson's equation is also used to model and calculate the electrostatic potential within the solar cell, accounting for the distribution of spatial charge density.^18^4
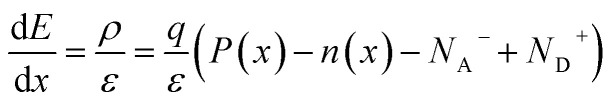
where *E* is the electric field, *ρ* is the charge density, *q* is the electric charge, *ε* is the material permittivity, *n*, *P* are the electron and hole concentration, respectively and *N*_A_, *N*_D_ are acceptor and donor carrier concentrations, respectively. The cell structure is presented in Fig. 1a while the corresponding energy band alignment is given in Fig. 1b. Further, the basic input parameters are shown in Table 1.

**Table 1:** Basic input parameters of the proposed optimized solar cell

Parameter	Spiro-OMeTAD^4,19^	N719 dye^20^	PC61BM^21^	TiO_2_ (ref. 4)	FTO^22–24^
Thickness, (nm)	50.00	2000.0	50.0	30.0	100.0
Bandgap, *E*_g_, (eV)	2.91	1.6	2.1	3.2	3.5
Electron affinity, *χ*, (eV)	2.2	3.9	3.9	4	4
Dielectric permittivity, relative, (*ε*_r_)	3.0	30	3.9	9.0	9.0
CB effective density of states, *N*_c_, (cm^−3^)	2.8 × 10^19^	2.4 × 10^20^	2.2 × 10^19^	2.0 × 10^18^	2.2 × 10^18^
VB effective density of state, *N*_v,_ (cm^−3^)	1.0 × 10^19^	2.5 × 10^20^	2.2 × 10^19^	1.8 × 10^19^	1.8 × 10^19^
Electron mobility, *µ*_n_, (cm^2^ V^−1^ S^−1^)	1.2 × 10^−4^	5.0 × 10^0^	2.0 × 10^−1^	2.0 × 10^1^	2.0 × 10^1^
Hole mobility, *µ*_p_ , (cm^2^ V^−1^ S^−1^)	2.0 × 10^−4^	5.0 × 10^0^	2.0 × 10^−1^	1.0 × 10^1^	1.0 × 10^1^
Density of n-type doping, *N*_D_, (cm^−3^)	0.0 × 10^0^	1.0 × 10^17^	1.0 × 10^−3^	0.0 × 10^0^	2.0 × 10^19^
Density of p-type doping, *N*_A_, (cm^−3^)	2.0 × 10^18^	1.0 × 10^7^	2.0 × 10^−3^	9.0 × 10^16^	0.0 × 10^0^
Defect density, *N*_t_, (cm^−3^)	1.0 × 10^15^	1.0 × 10^12^	0.0 × 10^0^	0.0 × 10^0^	1.0 × 10^15^

## 3 Results and discussion

### 3.1 Absorption characteristics of the proposed solar cell architecture

The absorption spectra of the different layers in a solar cell are fundamental to its performance. The active layer, which is typically a semiconductor like N719 dye has a specific bandgap that dictates the range of light it can absorb.^25^ Only photons with energy at or above the bandgap are absorbed, creating electron–hole pairs that generate current. Photons with less energy pass right through. To maximize the amount of sunlight captured, solar cells especially thin-film and multijunction types often use multiple layers, each designed to absorb a different part of the solar spectrum.^26^ Ultimately, a solar cell's efficiency hinges on how well the combined absorption spectra of all its layers align with the sun's spectrum, allowing for maximum power generation. The optical absorption coefficient can be set up on a model equation as expressed by eqn (5):^18^5
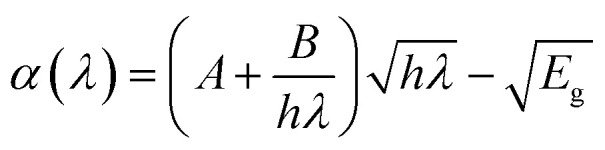
where *h* is Planck's constant, *E*_g_ is the bandgap energy, and *λ* is the wavelength of the incident light and *α* is the absorption Coefficient, which is expressed by eqn (6):^18^6
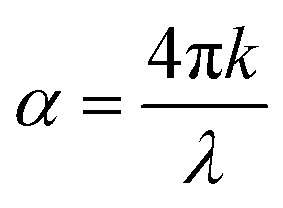


**Fig. 2:**
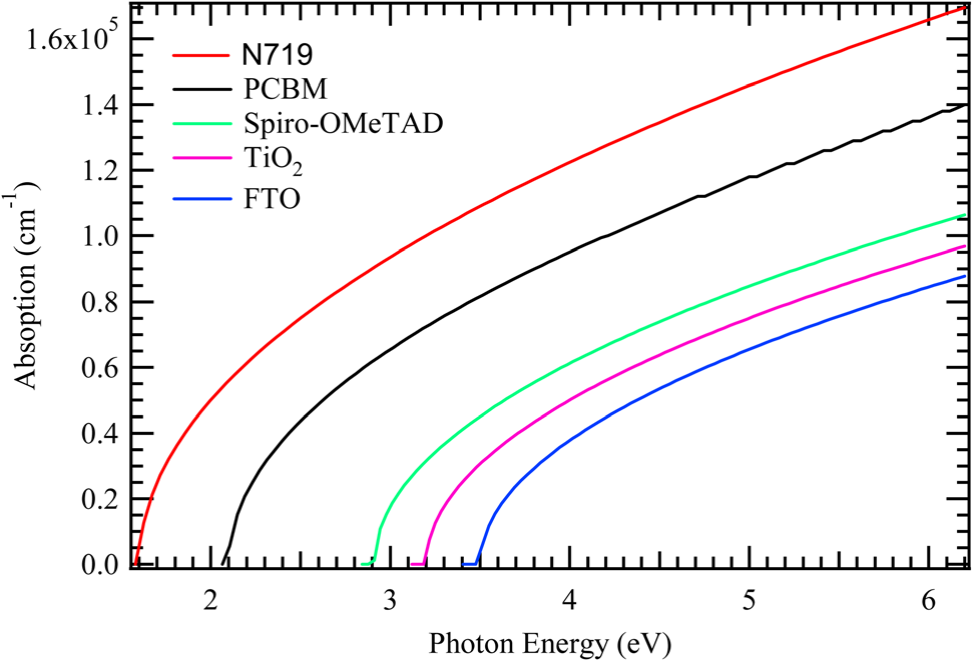
The absorption model of the cell structure.

Fig. 2 illustrates the absorption spectra of various layers within these solar cell structure, highlighting how each material interacts with incident light across the UV-visible region. This analysis is fundamental to the absorption model of solar cells, which evaluates the distribution and efficiency of light absorption across different layers in a multilayered device. Among the materials shown, the N719 dye exhibits the highest absorption, particularly across the visible spectrum, with strong absorbance from approximately 350 nm to 750 nm. This confirms its role as the primary light-harvesting material in DSSCs. The dye effectively captures photons and facilitates the generation of photoexcited charge carriers, making it the central contributor to photocurrent generation in the device.

In contrast, PCBM shows minimal absorption throughout the entire wavelength range, suggesting that it does not significantly contribute to light absorption. Instead, its function in the device is primarily electrical, it acts as an efficient electron transport material. Similarly, Spiro-OMeTAD, although showing slightly higher absorption than PC61BM, does not absorb light strongly enough to serve as a primary absorber. It plays a crucial role as a hole transport material, aiding in charge separation and transfer rather than light harvesting. The TiO_2_ layer begins to absorb in the ultraviolet region, particularly below 400 nm. This is consistent with its wide bandgap (3.2 eV), which limits its absorption to higher-energy UV photons. The negligible absorption by FTO is desirable, as it ensures minimal optical losses at the front interface.

### 3.2 Impact of *J*–*V* and QE characteristics

The *J*–*V* curve is a fundamental tool for evaluating the performance of a solar cell under illuminated conditions.^27^ On the other hand, QE describes how efficiently a solar cell converts incoming photons into electrical current. It is usually divided into external quantum efficiency (EQE), which accounts for all incident photons, and internal quantum efficiency (IQE), which considers only the photons that are actually absorbed by the active layer.^28^ A high QE indicates effective light absorption and charge carrier collection, while low values can point to issues such as poor absorption, recombination, or interface defects.

**Fig. 3:**
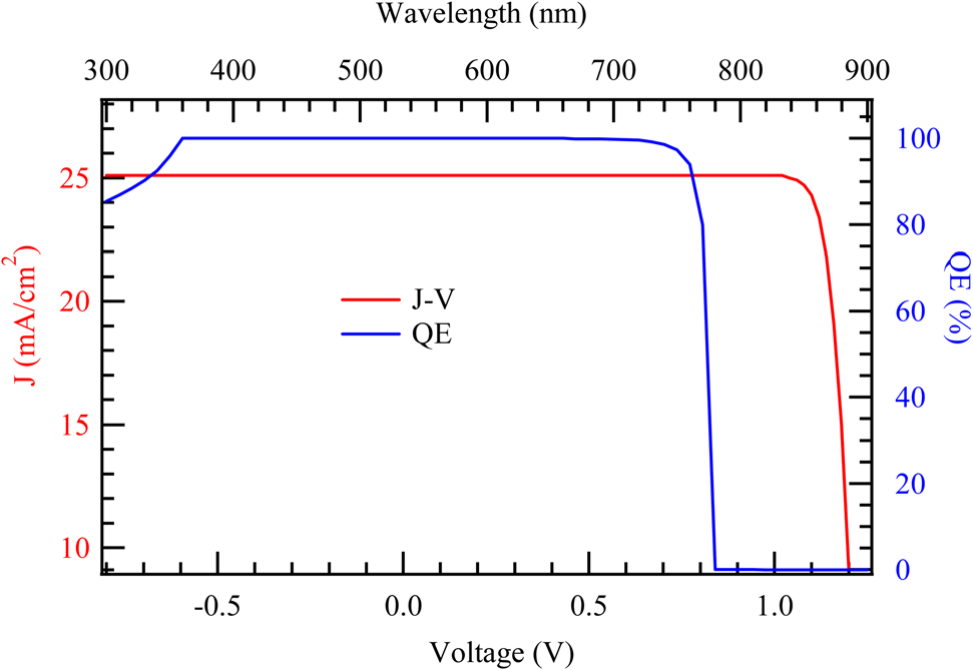
*J*–*V* and QE characteristics for the cell architecture.

Fig. 3 depicts the *J*–*V* and QE characteristics of the simulated solar cell architecture. The *J*–*V* curve exhibits a nearly ideal rectangular profile, indicative of a high-performance device with minimal recombination losses and efficient charge transport. The current density reaches approximately 25 mA cm^−2^ at zero voltage, while the voltage extends to 1.2 V, where the current density approaches zero. The shape of the *J*–*V* curve suggests a high FF, further confirming the device's excellent electrical performance. Collectively, these parameters imply a high PCE, demonstrating the effectiveness of the solar cell's design and material configuration. The QE curve, shown in blue, reflects the device's external quantum efficiency across a range of wavelengths. The quantum efficiency remains consistently above 90% throughout the visible region, approximately between 350 nm and 800 nm, indicating highly efficient photon absorption and charge carrier collection across the active spectrum. The steep decline beyond 800–850 nm marks the cutoff wavelength and signifies the optical bandgap of the absorber material, which is likely around 1.6 eV. Additionally, the gradual drop in QE at shorter wavelengths may be attributed to surface recombination or optical losses near the front interface.

### 3.3 Variation of absorber thickness with electrical outcomes of the cell

The thickness of the absorber layer in a solar cell plays a crucial role in determining its performance, particularly in terms of *J*_sc_ and QE, which in turn influence key electrical parameters such as *V*_oc_, FF, and overall PCE. When the absorber layer is too thin, it fails to absorb a sufficient number of photons, particularly those with longer wavelengths that penetrate deeper into the material.^29^ This results in a lower *J*_sc_ because fewer charge carriers are generated. Additionally, thin absorbers are more susceptible to surface recombination losses, where charge carriers recombine at the interfaces before being collected, further reducing both QE and *J*_sc_.^30^ However, very thin absorbers can sometimes exhibit a slightly higher *V*_oc_ if bulk recombination is minimized, though this advantage is often outweighed by the significant loss in current. As the absorber thickness increases, more photons are absorbed, leading to a rise in *J*_sc_ and QE, particularly in the long-wavelength region where absorption was previously weak.^31^

**Fig. 4:**
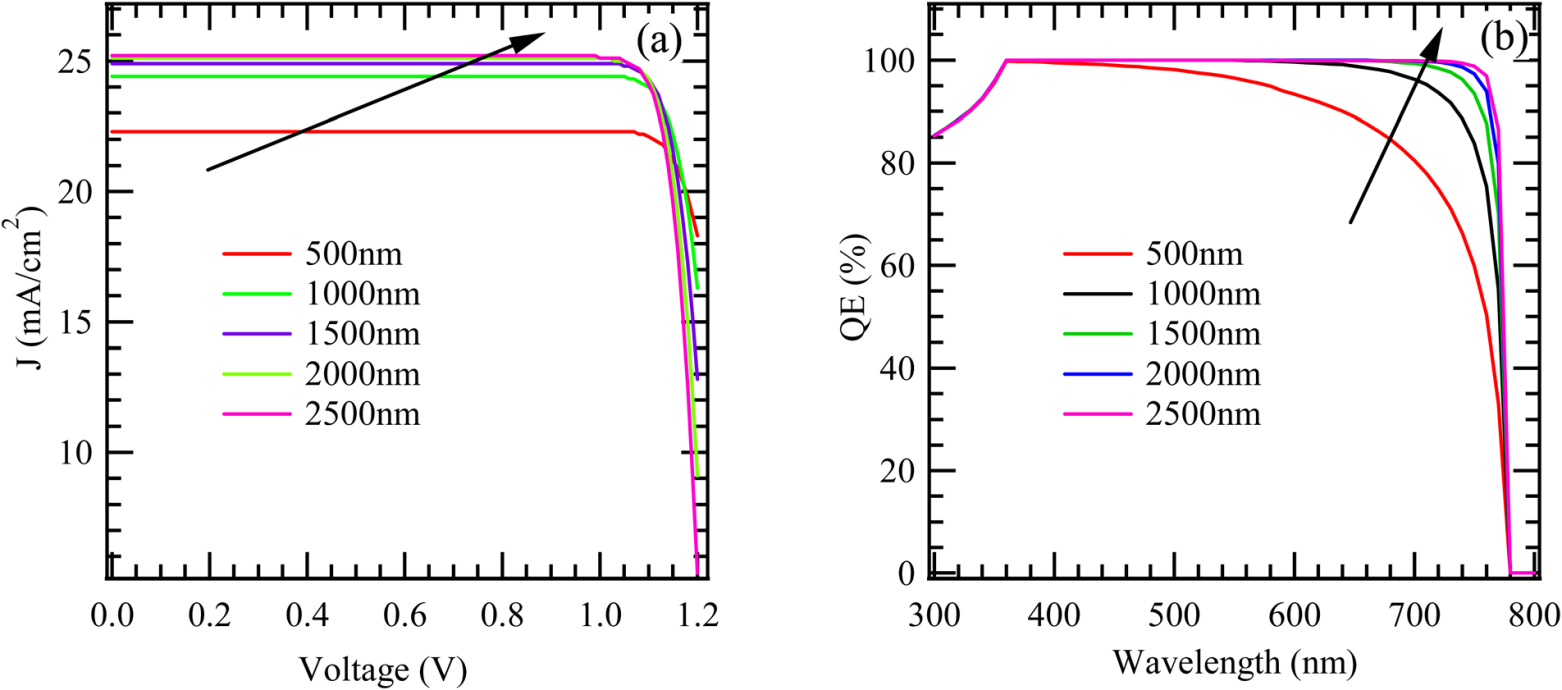
Variation of *J*–*V* and QE characteristics with the thickness of the N719.

Fig. 4a illustrates the variation in *J*–*V* characteristics of the solar cell as a function of the absorber layer thickness, ranging from 500 nm to 2500 nm. The graph reveals that increasing the absorber thickness leads to a notable enhancement in the *J*_sc_. This improvement is attributed to the greater optical path length in thicker absorber layers, which allows for more efficient light absorption and, consequently, the generation of more photocarriers. Among the curves, the device with a 500 nm absorber exhibits the lowest *J*_sc_, while the one with a 2500 nm absorber achieves the highest, indicating a direct relationship between thickness and photogeneration up to a certain point.

In contrast, the *V*_oc_ remains relatively stable across the various thicknesses. This suggests that the built-in potential and recombination mechanisms near the junction are not significantly affected by changes in absorber thickness within the range studied. Although minor variations in *V*_oc_ may occur due to increased recombination in very thick layers, these effects appear to be negligible here. The FF also shows a slight improvement with increasing thickness, particularly between 500 nm and 1500 nm. This enhancement could be due to improved charge collection efficiency and reduced series resistance in thicker absorbers. However, beyond a certain thickness around 2000 nm the FF appears to plateau or marginally decrease, likely due to increased bulk recombination or carrier transport limitations in excessively thick layers. As a result of the combined behavior of *J*_sc_, *V*_oc_, and FF, the PCE increases with absorber thickness, reaching its peak in the range of 1500 to 2000 nm. Beyond this point, the efficiency gain diminishes, indicating the onset of saturation where further thickness does not significantly improve performance and may even introduce parasitic losses.

Overall, the *J*–*V* characteristics in Fig. 4b suggest that absorber layer thickness is a critical parameter in optimizing device performance. While thinner absorbers suffer from limited light absorption, overly thick ones may face charge transport challenges. Thus, an intermediate thickness, particularly around 1500–2000 nm, offers an optimal balance between light harvesting and charge collection. Fig. 3b illustrates the QE response of the solar cell as a function of absorber layer thickness, with thicknesses varying from 500 nm to 2500 nm. The QE spectrum reflects the cell's effectiveness in converting incident photons into charge carriers across different wavelengths. A clear trend emerges, where increasing the absorber thickness leads to an overall enhancement in QE, particularly in the longer wavelength region of the spectrum.

For the device with a 500 nm thick absorber layer, the QE begins to decline significantly beyond 600 nm. This is due to insufficient absorption of lower-energy photons, which penetrate deeper into the material. Thinner layers are unable to effectively absorb these photons, leading to lower carrier generation in the red and near-infrared regions. As the thickness increases to 1000 nm and beyond, the QE in this spectral range improves markedly, indicating more efficient absorption and photogeneration. By the time the absorber reaches 2000 nm and 2500 nm, the QE response has nearly saturated, suggesting that most of the usable light is absorbed and converted in layers of these thicknesses. In the short-wavelength region, from approximately 300 nm to 500 nm, the QE curves for all thicknesses show relatively similar behavior. This suggests that high-energy photons are predominantly absorbed near the surface of the absorber, and therefore thickness has a limited influence on their collection. However, slight variations still exist, with the thinnest absorber showing marginally lower QE, likely due to surface recombination or incomplete absorption.

### 3.4 Temperature variation with electrical outcomes

The extent of this temperature-induced efficiency loss is measured by the temperature coefficient, which indicates the percentage decrease in performance per degree Celsius above the standard operating temperature (typically 300 K).^32^ For most silicon solar cells, efficiency declines by approximately 0.3% to 0.5% for every 1 °C rise.^33^ Prolonged exposure to high temperatures can also lead to material degradation, shortening the lifespan of photovoltaic panels. To counteract these effects, cooling strategies such as improved ventilation, heat-dissipating materials, or active cooling systems are often implemented. A thorough understanding of temperature effects is essential for optimizing solar panel placement and design, ensuring consistent energy production across different environmental conditions.

**Fig. 5:**
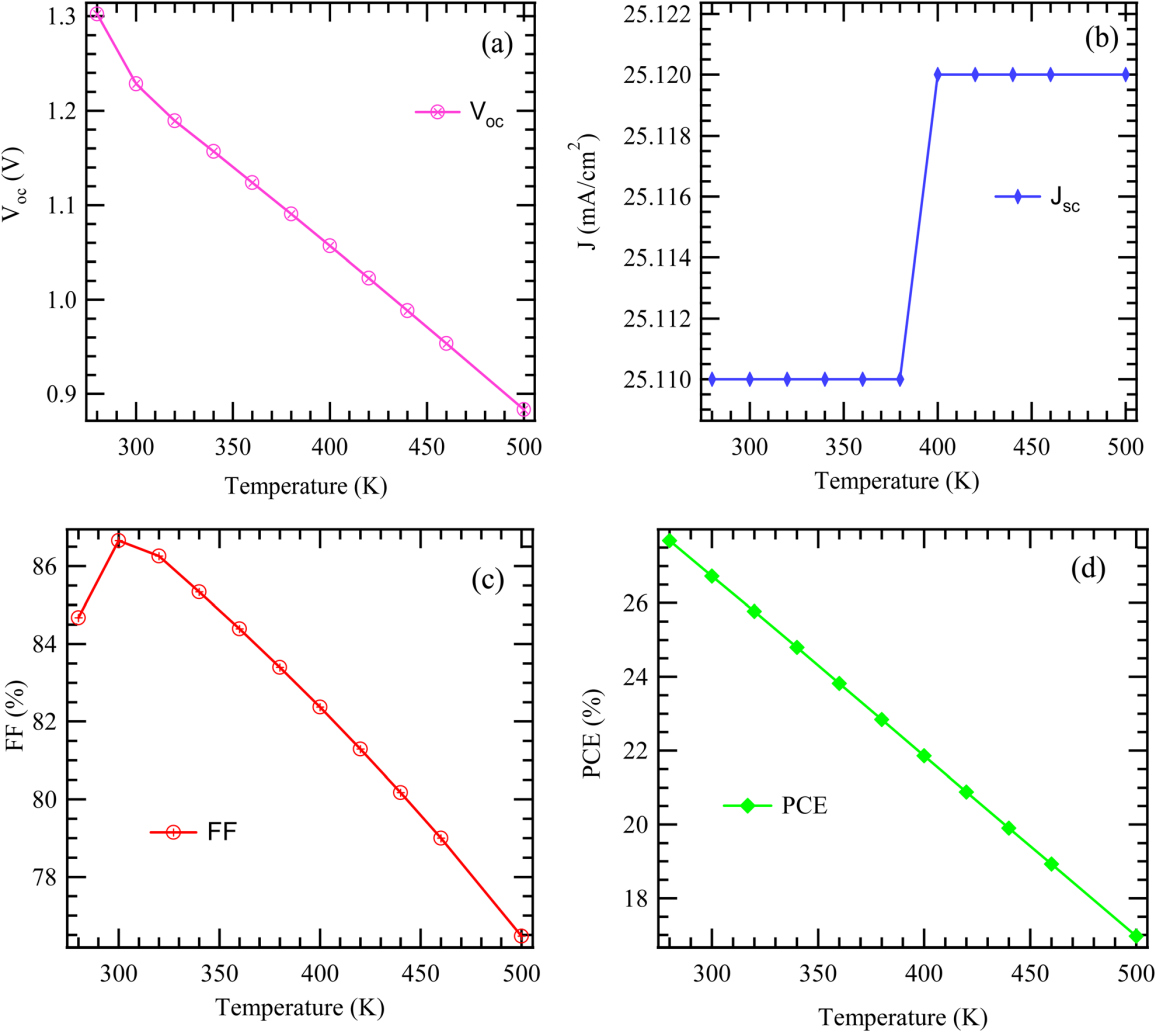
Variation of temperature with photovoltaic parameters.

Fig. 5a depicts the variation of the *V*_oc_ of a solar cell as a function of temperature. As the operating temperature rises from around 280 K to 500 K, there is a clear and steady decline in *V*_oc_. This inverse correlation is a well-established behavior in solar cells. The open-circuit voltage depends on both the bandgap energy of the material and the thermal voltage; the latter being directly influenced by temperature. The primary cause of the *V*_oc_ reduction with temperature is the exponential increase in the reverse saturation current (*I*_0_), which is highly temperature-sensitive. According to the relation7
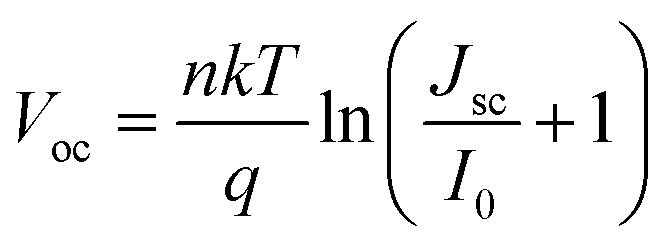


Fig. 5c illustrates the variation in the FF of a solar cell as a function of operating temperature. As the temperature increases from around 280 K to approximately 300 K, the FF experiences a modest rise, peaking slightly above 86%. However, beyond this point, a distinct downward trend emerges. With further temperature increase, the fill factor steadily and significantly declines, reaching a value below 78% at 500 K. This behavior is typical of solar cells and is mainly due to the temperature-induced reduction in *V*_oc_ and the increase in series resistance (*R*_s_). Although the *J*_sc_ may exhibit a slight improvement, the negative effects of decreasing *V*_oc_ and increasing *R*_s_ outweigh it, resulting in a notable drop in the fill factor. Consequently, this reduction in FF contributes to the overall decline in power conversion efficiency at higher temperatures.

As temperature increases, *I*_0_ increases significantly, resulting in the observed drop in *V*_oc_. This trend underscores the critical role of thermal regulation in sustaining solar cell efficiency, since elevated temperatures diminish the maximum achievable power output. Fig. 5b illustrates how the *J*_sc_ of the solar cell varies with temperature. The plot demonstrates a non-linear trend. Between approximately 280 K and 380 K, *J*_sc_ remains nearly constant at around 25.110 mA cm^−2^. Beyond 380 K, however, there is a sudden and pronounced rise, reaching a new stable level of approximately 25.120 mA cm^−2^, which persists up to 500 K. This sharp transition contrasts with the typical behavior of conventional silicon solar cells, which generally exhibit a gradual, linear increase in *J*_sc_ with temperature due to bandgap narrowing. The abrupt shift observed here may point to a phase transition or a change in the material's physical properties around 380 K. Such a discontinuity is characteristic of specialized materials such as phase-change compounds or advanced semiconductor architectures where specific thermally activated mechanisms come into play.

Fig. 5d illustrates the correlation between a solar cell's PCE and its operating temperature. A pronounced inverse linear relationship is observed, with PCE steadily decreasing as temperature rises from around 280 K to 500 K. Specifically, the efficiency drops from over 26% to just under 17%. This decline is primarily attributed to temperature-induced variations in key performance parameters, namely the *V*_oc_ and the FF. Since PCE is directly proportional to the product of *V*_oc_, the *J*_sc_, and FF, as given by the equation PCE = (*V*_oc_·*J*_sc_·FF)/*P*_in_, any significant reduction in *V*_oc_ and FF leads to a notable drop in overall efficiency. Although *J*_sc_ may show a slight increase with temperature, it is insufficient to counterbalance the losses in *V*_oc_ and FF. These findings highlight the essential role of thermal management in solar cell design to ensure optimal performance and durability.

### 3.5 Mott–Schottky characteristics

Mott–Schottky (M–S) analysis is a fundamental technique used to investigate the electrical properties of solar cells by examining their capacitance–voltage (*C*–*V*) characteristics.^34^ The Mott–Schottky plot, which graphs 1/*C*^2^ as a function of applied voltage, provides essential parameters such as built-in potential (*V*_bi_), doping concentration (*N*_D_ or *N*_A_), and depletion layer width.^35^ Non-linearities in the plot often point to interface defects or surface states that can hinder efficiency. The built-in potential (*V*_bi_) of the solar cell can be estimated from the Mott–Schottky plot according to eqn (8).^36^8
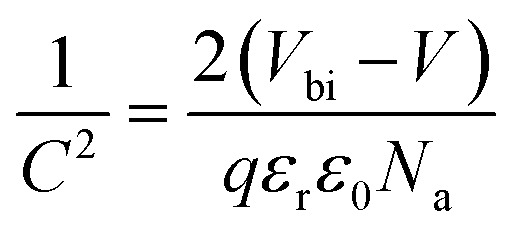
where, *C* is the capacitance, *V*_bi_ is the built-in potential, *V* is the applied voltage, *q* is the charge of the carrier, *ε*_r_ is the relative permittivity, *ε*_0_ is the permittivity of the free space and *N*_a_ is the doping density.

The depletion width (Wd) can be calculated using eqn (9) as a function of applied voltage.^36^9
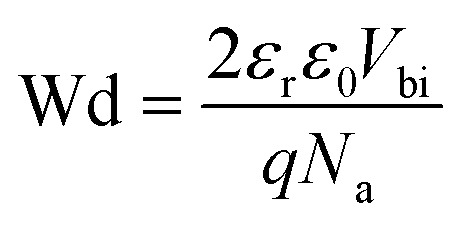


The technique also plays a crucial role in advancing next-generation photovoltaic technologies, including perovskite and organic solar cells. For perovskite-based devices, MS analysis helps probe ion migration and interfacial charge dynamics, which contribute to hysteresis and stability issues.^37^ By assessing voltage-dependent capacitance, researchers can identify trap states, doping irregularities, and degradation pathways that impact charge extraction. Additionally, MS measurements aid in characterizing energy band alignment at critical interfaces, such as between the light-absorbing layer and charge transport materials.^38^

**Fig. 6:**
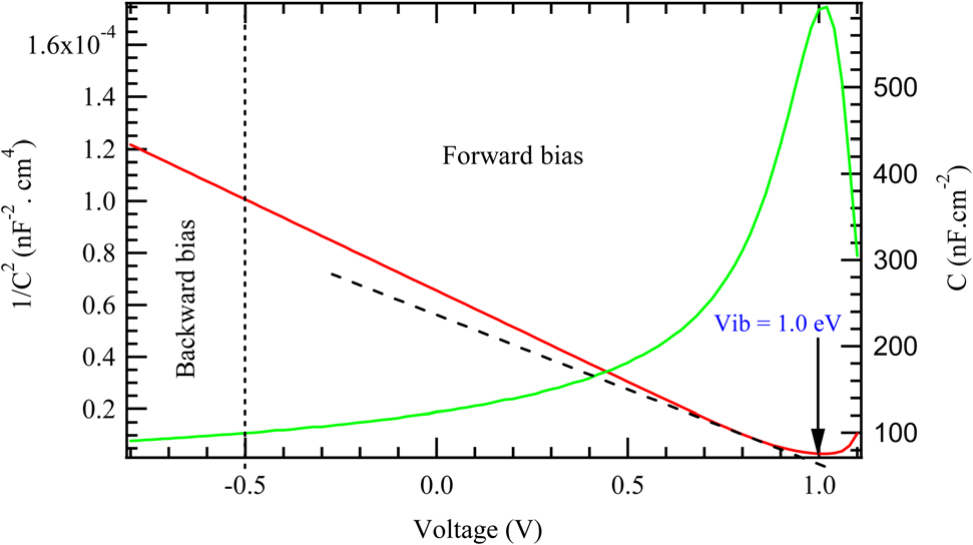
M–S characteristics of the model cell.

Fig. 6 presents the M–S characteristics of the model solar cell, where the inverse square of the capacitance (1/*C*^2^) is plotted against the applied voltage. This technique is widely used to investigate key electrical properties of solar cells, particularly the built-in potential and doping characteristics of the absorber layer. In the reverse bias region (represented by the red curve), the plot exhibits a linear trend, which is characteristic of a well-defined depletion region. This linear segment is crucial for extracting the *V*_bi_, which is determined from the *x*-axis intercept of the extrapolated linear fit. From the graph, the built-in potential is estimated to be approximately 1.0 eV. A high built-in potential typically correlates with a high *V*_oc_, suggesting efficient charge separation and favorable junction properties within the solar cell.

The forward bias region shows a marked deviation from linearity. As the applied voltage increases, the capacitance initially rises due to the narrowing of the depletion region. However, beyond a certain point, the curve peaks and then declines, indicating the influence of non-ideal effects. This nonlinear behavior in forward bias is commonly associated with factors such as high-level carrier injection, the presence of interface states or traps, series resistance, and possible ionic migration especially in perovskite or thin-film devices. The slope of the linear region in the reverse bias regime can also be used to estimate the doping concentration of the absorber layer, based on the Mott–Schottky relation. A steeper slope corresponds to a lower doping density, while a gentler slope indicates a higher level of doping. These insights are essential for understanding the internal electric field distribution and optimizing the device's electrostatic properties.

### 3.6 Generation and recombination

The efficiency of solar cells fundamentally depends on the delicate balance between charge carrier generation and recombination processes. When sunlight interacts with the semiconductor material, photons with energy exceeding the material's bandgap excite electrons from the valence band to the conduction band, creating electron–hole pairs in a process called photogeneration.^39^ This initial step determines the maximum possible current the device can produce. However, not all generated carriers contribute to the useful output, as various recombination mechanisms continuously work to annihilate these charge carriers before they can be collected at the electrodes. Modern solar cell designs focus on engineering solutions to maximize generation while minimizing these recombination losses. This involves optimizing light absorption through anti-reflective coatings and light-trapping structures, while simultaneously reducing recombination through high-quality material growth, effective passivation of surfaces and interfaces, and careful control of doping concentrations.

**Fig. 7:**
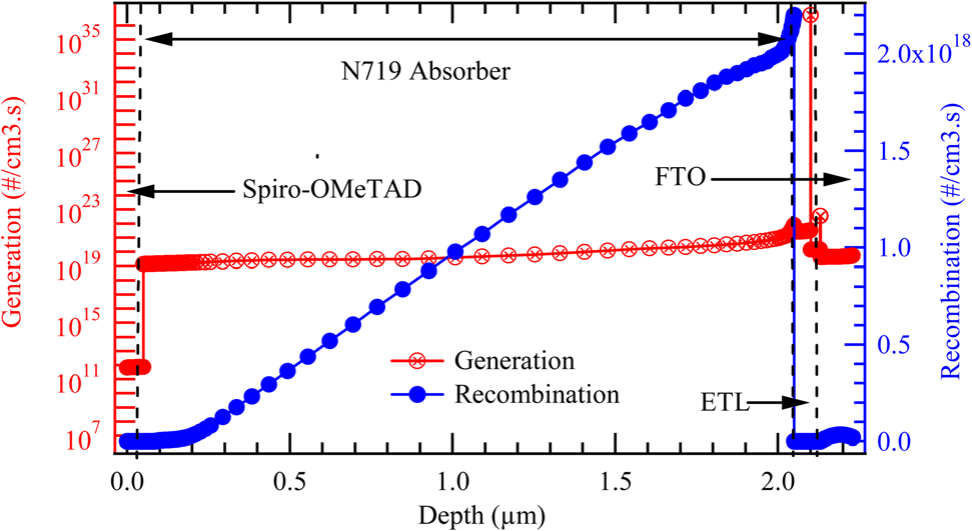
Generation-recombination characteristics of the cell structure.

Fig. 7 presents the spatial distribution of carrier generation and recombination rates across the thickness of the simulated solar cell. The curve, plotted on the left logarithmic axis, represents the generation rate (in #/cm^3^ s), while the curve on the right logarithmic axis shows the recombination rate (in #/cm^3^ s) as a function of depth within the device structure. From the plot, it is evident that the generation rate remains relatively constant at approximately 10^19^ cm^−3^ s^−1^ throughout the active absorber layer. This uniformity suggests consistent absorption of photons and efficient generation of charge carriers across the bulk of the absorber. However, slight deviations are observed near the front and rear interfaces, particularly at the back contact, where the generation rate drops slightly but still higher than the recombination rate. This may be attributed to limited light absorption near the rear interface or optical losses due to reflection or parasitic absorption in the adjacent layers.

In contrast, the recombination profile shows a more dynamic trend. It begins at very low values around 10^7^ cm^−3^ s^−1^near the front interface, gradually increases through the absorber, and peaks sharply near the rear interface, reaching a maximum of approximately 2.0 × 10^18^ cm^−3^ s^−1^. This steep peak in recombination near the back contact indicates a region of significant carrier loss, likely caused by high defect density, poor energy band alignment, or the presence of interfacial trap states that facilitate non-radiative recombination. When comparing generation to recombination across the device, the generation-to-recombination (*G*/*R*) ratio offers important insights into device performance. Near the front contact, the *G*/*R* ratio is extremely high on the order of 10^12^ indicating that virtually all photogenerated carriers survive recombination and contribute to current. In the middle of the absorber, the G/R ratio remains favorable (around 10^4^), further confirming the high-quality nature of the absorber material. However, toward the rear interface, this ratio drops dramatically, approaching values below 1.0. This implies that recombination becomes comparable to, or even exceeds, generation in this region, severely limiting carrier collection efficiency. Generally, To improve device efficiency, it is essential to mitigate recombination at this interface. Strategies such as passivating the rear contact, introducing buffer layers, or optimizing the work function and band alignment of the back electrode can help suppress recombination and enhance overall device performance.

### 3.7 Impact of band alignment diagram on cell performance

The band alignment diagram is a key tool for understanding and improving solar cell performance, especially in devices with multiple layers.^40^ This diagram illustrates the energy levels of different materials where they meet, which directly controls how electrons and holes move. The ideal setup, known as a staggered or type-II alignment, allows photogenerated electrons and holes to be efficiently separated and sent to their respective electrodes.^41^ Poor alignment, such as a “cliff-like” offset, creates a barrier that blocks carrier movement, causing them to recombine at the interface. This significantly reduces the cell's *J*_sc_ and *V*_oc_. On the other hand, a “spike-like” offset can cause carriers to move backward, also leading to recombination and limiting efficiency. Therefore, carefully choosing materials with the right electron affinities and bandgaps to achieve the correct band alignment is critical for maximizing a solar cell's PCE.

**Fig. 8:**
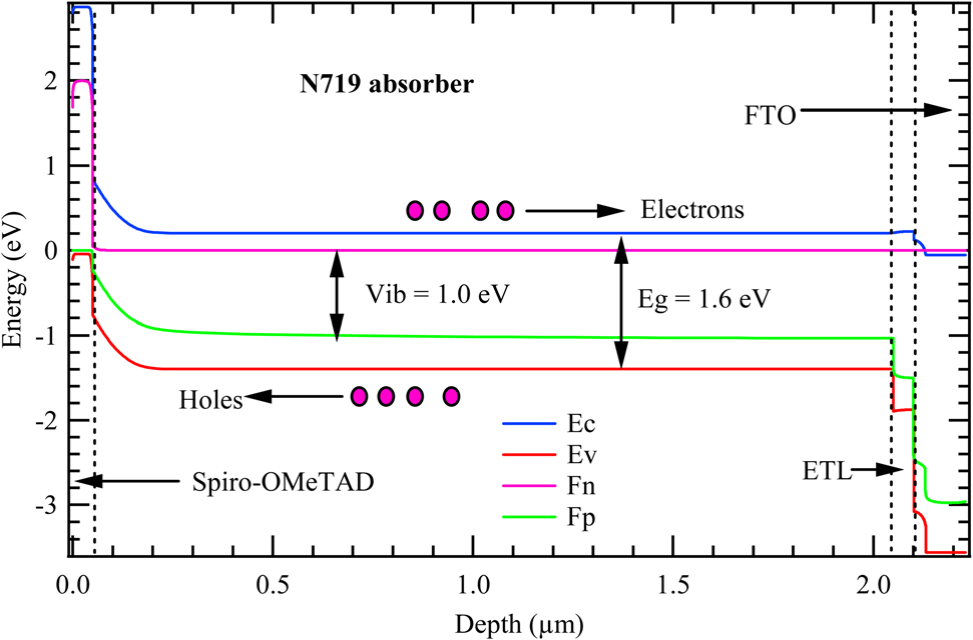
Band alignment energy diagram of the model cell.

Fig. 8 presents the band alignment energy diagram of the model solar cell, illustrating the internal energy landscape across the device under illumination. Four primary energy curves are shown: the conduction band minimum (*E*_c_), valence band maximum (*E*_v_), the electron quasi-Fermi level (*F*_n_), and the hole quasi-Fermi level (*F*_p_). These curves collectively describe how charge carriers behave and how potential differences develop across the cell. The solar cell model is composed of several key layers, beginning on the left with Spiro-OMeTAD, HTL, followed by the N719 dye absorber, which dominates the central region, and then the ETL on the right. The far-right edge is occupied by the FTO layer, which serves as the transparent window. In the absorber layer (N719), the bandgap is clearly defined by the energy difference between the valence band maximum and conduction band minimum, measured at 1.6 eV, which determines the spectral range of light that the material can absorb. When photons with energy equal to or greater than this bandgap are absorbed, electrons are excited from the valence band to the conduction band, creating electron–hole pairs. These charge carriers are then spatially separated due to the built-in electric field: electrons move toward the ETL and ultimately to the FTO, while holes are transported in the opposite direction toward the HTL (Spiro-OMeTAD).

The quasi-Fermi levels *F*_n_ and *F*_p_ split under illumination indicating a non-equilibrium condition essential for power generation. The magnitude of this splitting in the absorber layer represents the maximum photovoltage that can be achieved, commonly *V*_oc_. In this case, the *F*_n_ and *F*_p_ levels are separated by approximately 1.0 eV. The plot implies the presence of a built-in potential (*V*_bi_) of 1.0 eV which aligns with the quasi-Fermi level separation. The relatively high built-in potential in the current cell is very important in enhancing charge injection and increased device efficiency.^42^

## 4 Impedance spectroscopy analysis

**Fig. 9:**
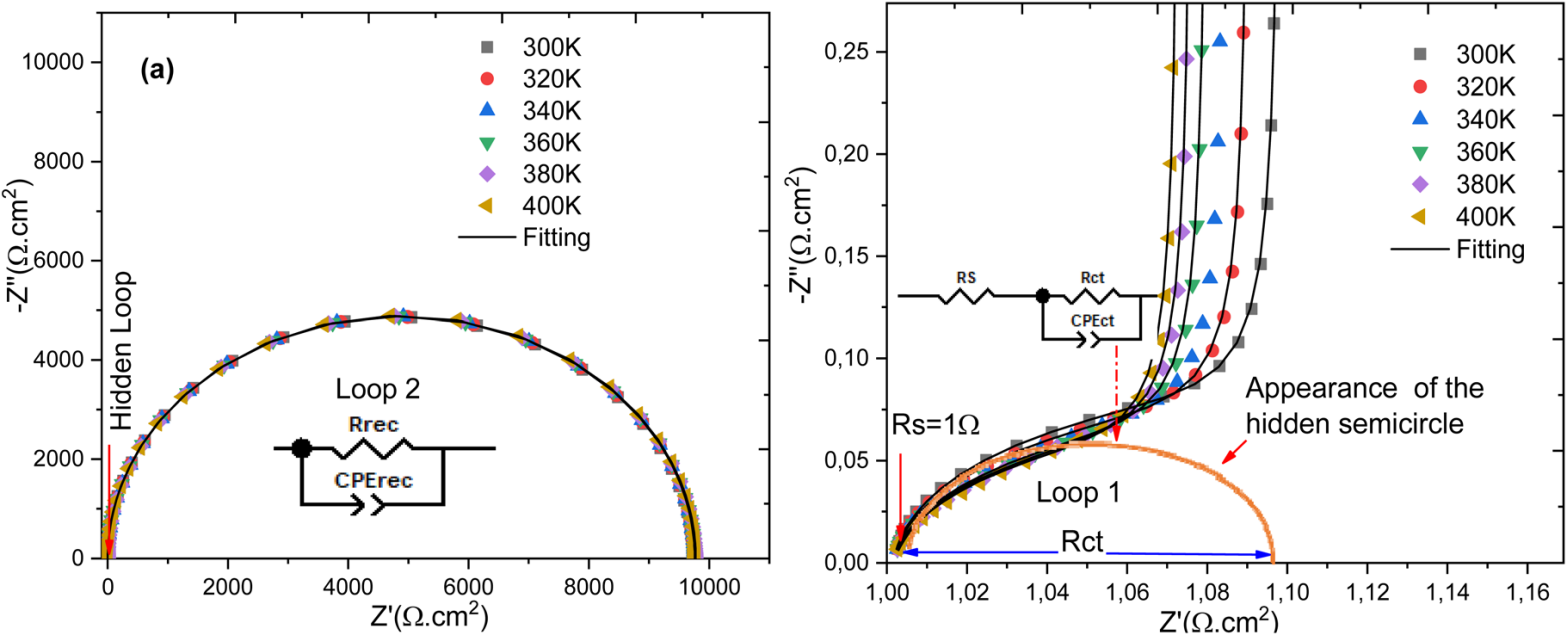
Nyquist plot for all temperatures studied (a) and (b) Nyquist plot with a high-frequency zoom-in.

The Nyquist plot aims to highlight the number of semicircles present, with each semicircle representing a characteristic relaxation time. According to Fig. 9 of the Nyquist plot, a single dominant loop is observed, associated with the recombination resistance and the recombination capacitance. In order to demonstrate that there are two distinct relaxation times one corresponding to the charge transfer process and the other to the recombination process we will use electrical conductivity. This will allow us to determine the resistance related to charge transfer as well as that associated with recombination. The Nyquist plot is a graphical representation used to analyze electrochemical properties and transport phenomena in materials. Each semicircle on this plot corresponds to a relaxation time, representing a characteristic delay associated with a specific physical process, such as diffusion, recombination, or charge transfer.^43,44^

In the analysis conducted, a single dominant loop is observed in a frequency range of 1 mHz to 40 MHz, suggesting that in the material or device under study, recombination resistance and recombination capacitance are the predominant phenomena. This behavior is typical when the recombination of charge carriers is the limiting process in the system.^45,46^ On the other hand, charge transfer occurs at higher frequencies, between 40 MHz and 1 GHz, and is related to the material's ability to allow the circulation of electrons or holes. Recombination, on the other hand, involves the loss of charge carriers that recombine before contributing to current transport.^47,48^

**Fig. 10:**
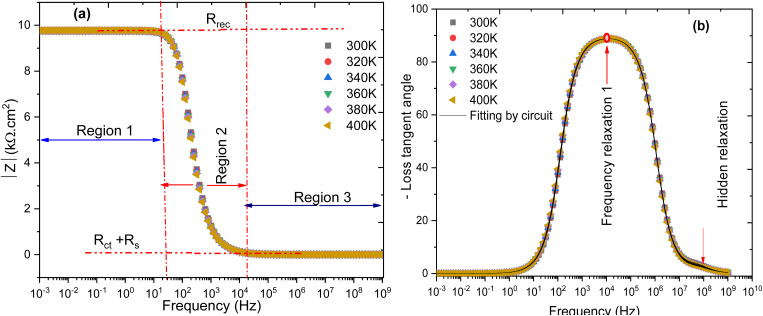
Behavior of the complex impedance modulus and loss tangent as a function of temperature.

Fig. 10 shows the evolution of the complex impedance modulus (|*Z*|) and the loss tangent (tan *δ*) as a function of temperature, within the range of 300 K to 400 K. These results provide detailed insight into the electrochemical behavior of the studied cell. On the left side of the figure, the impedance modulus displays three distinct frequency regions. In the [1 mHz–100 Hz] range, |*Z*| remains constant at approximately 9749 Ω cm^2^ across all temperatures, indicating a high-impedance regime, likely related to slow processes such as interfacial recombination or limitations in ionic transport. In the intermediate frequency range [100 Hz–10 kHz], a significant drop in impedance is observed, reflecting a transition toward a conduction-dominated regime. Finally, in the [10 kHz–1 GHz] region, the modulus stabilizes around 1.00749 Ω cm^2^, suggesting a purely ohmic response associated with the series resistance of the device, as supported by recent studies on thin-film solar cells.^49,50^

Meanwhile, the loss tangent (tan *δ*) remains consistently negative and nearly overlapping across all temperatures, indicating a predominantly capacitive behavior of the system. The thermal invariance of tan *δ* also implies that dissipative mechanisms such as resistive losses or non-radiative recombination are not significantly affected by temperature within the studied range. This behavior is typical of thermally stable systems, where both interfaces and active layers exhibit low thermal sensitivity, as observed in perovskite and stable organic photovoltaic devices.^51,52^

**Fig. 11:**
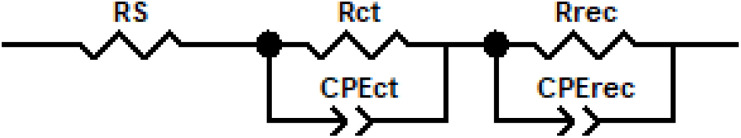
Equivalent electrical representation of a solar cell's.

After analyzing the Nyquist plot with Zview version 2.2 software to explore the dielectric functions, the goal was to determine the optimal equivalent electrical circuit that replicates the observed impedance spectra. The circuit that models the cell's behavior over the used frequency range consists of a series resistance, *R*_s_, representing the front and rear contacts, along with resistance *R*_ct_ and CPET_ct_ that form the branch modeling the charge transfer process. Additionally, resistance *R*_rec_ and CPET_rec_ represent the recombination process. By applying the principle of superposition, the electrical behavior of the solar cell conversion is represented by the equivalent circuit shown in Fig. 11.

### 4.1 Cole–Cole relaxation theory and Jonscher's universal power law for electrical conductivity

**Fig. 12:**
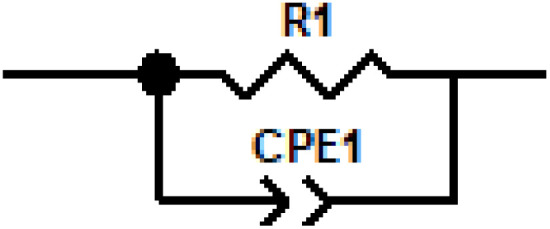
Cole–Cole relaxation circuit.

This circuit represents the Cole–Cole relaxation in Nyquist diagram^53^ as shown in Fig. 12.

The expression for the Cole–Cole impedance is as follows:10
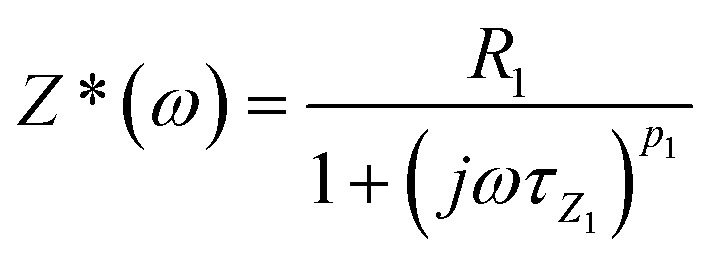


Therefore, the expression for the complex impedance (Z*) is found to be analogous to the one previously given in 68.

The decomposition of the complex impedance (Z*) into its real and imaginary components yields the following expressions:11
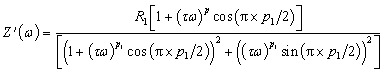
12
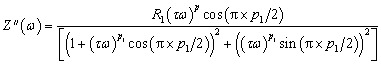


From a theoretical perspective, the critical angular frequency corresponding to the peak of the imaginary part of the impedance, *Z*″(*ω*), can be determined by taking the derivative of *Z*″(*ω*) with respect to angular frequency and solving the following condition: d*Z*″(*ω*)/d*ω* = 0. In this case, the angular frequency at which the imaginary part Z″(*ω*) reaches its maximum was found to be as follows:

13 *ω*_max1_ = 1/*τ* = 1/(*RT*)^*p*_1_^

As a result, the complex impedance Z*(*ω*) can be expressed by the following equation, which is analogous to the one used to represent the Cole–Cole relaxation process.^69^14
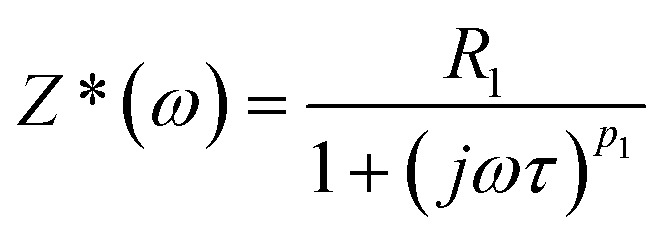


Here, *τ*_z_= (2π*F*)^−1^ = 1/*ω*_max_ represents the relaxation time, where *ω*_max_ is the angular frequency at which the imaginary part of the impedance reaches its maximum. The electrical strength is defined as Δ*Z* = *Z*_s_ − *Z*_∞_, where *Z*_∞_ = 0 corresponds to the impedance at high frequency, and *Z*_s_ = *R*_1_ represents the impedance at low frequency. The overall complex impedance Z*(*ω*) of the equivalent circuit shown in Fig. 14 can be expressed as the sum of the impedances of the sub-circuits corresponding to each individual block according to eqn (15).15
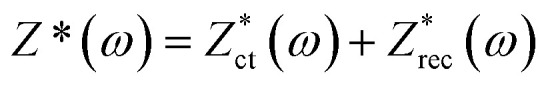


At high frequencies, the appearance of a semicircular arc in the Nyquist plot is commonly attributed to the charge transfer resistance within the device. In the specific layer sequence N719/PC61BM/TiO_2_/FTO, this response reflects the electron transfer pathway from the photoexcited N719 dye through PC61BM and TiO_2_, ultimately reaching the FTO electrode. This phenomenon is consistent with previous electrochemical impedance spectroscopy (EIS) analyses, which associate high-frequency semicircles with interfacial charge transfer processes at the electron transport layer (ETL) interface.^54^

At intermediate frequencies, the observed arc generally results from a superposition of interfacial and bulk processes, including ionic motion, dielectric polarization, and charge accumulation. These mechanisms have been widely identified as sources of non-ideal behavior in hybrid and dye-sensitized solar cells.^54,55^

For each semicircle observed, specific characteristic parameters were extracted at fixed measurement intervals:

(i). The resistance *R*_i_, obtained from the intercept of the semicircle with the real axis, corresponds to the dominant resistive contribution within that frequency domain.

(ii). The dispersion coefficient pi and pseudo-capacitance *T*_i_, derived from the fitting of a constant phase element (CPE), account for deviations from ideal capacitive behavior, often caused by interface roughness or non-uniform charge distribution.

(iii). The relaxation time *τ*_i_, indicating the characteristic time scale of the charge transport or polarization process, was calculated using eqn (12) in accordance with the methodology proposed by Chen *et al.*.^54^

The resistance associated with the recombination process is significantly influenced by temperature. Variations in the diameter of the semicircles across the Nyquist plots reflect systematic changes, highlighting the relative contributions of both charge transfer and recombination mechanisms. The values obtained from the fitted equivalent circuit model are summarized in Tables 2 and 3. Temperature significantly affects the performance of the solar cell, particularly by influencing the charge transfer resistance and the resistance associated with recombination processes. At room temperature, charge transfer occurs very rapidly, with a characteristic time about 3.11 ns, under electrical conditions where the series resistance is 1 Ω cm^2^ and the shunt resistance reaches 10 kΩ cm^2^.

**Table 2:** Parameters extracted from impedance modeling – charge transfer kinetic parameters

Temperature (K)	*R*_s_	*R*_ct_	CPE-P_ct_	CPE-T_ct_	Relaxation time of charge transfer (ns)
300	1.002	0.096985	0.95028	8.508 × 10^−8^	3.11628
320	1.002	0.089491	0.93631	1.159 × 10^−7^	2.97223
340	1.002	0.083647	0.92587	1.47 × 10^−7^	2.87516
360	1.002	0.078897	0.91876	1.761 × 10^−7^	2.80669
380	1.002	0.074924	0.91434	1.98 × 10^−7^	2.75427
400	1.002	0.071528	0.91195	2.152 × 10^−7^	2.7107

**Table 3:** Parameters extracted from impedance modeling – kinetics of charge recombination

Temp (K)	*R*_s_	*R*_rec_	CPE-P_rec_	CPE-T_rec_	Recombination relaxation time (ms)
300	1.002	9726	1	1.2504 × 10^−7^	1.216139
320	1.002	9739	1	1.2673 × 10^−7^	1.234223
340	1.002	9749	1	1.2856 × 10^−7^	1.253331
360	1.002	9759	1	1.3047 × 10^−7^	1.273257
380	1.002	9768	1	1.325 × 10^−7^	1.29426
400	1.002	9776	1	1.3466 × 10^−7^	1.316436

Table 4 summarizes the parameters extracted from the modeling of the influence of the relative permittivity (*ε*_r_) on the behavior of the N719 absorber. It includes, in particular, the values of quantum efficiency, open-circuit voltage, short-circuit current, and efficiency, as a function of variations in *ε*_r_. These results allow for the analysis of how the dielectric properties of the medium affect the performance of the N719 dye-sensitized solar cell. The Nyquist diagram shows a profile similar to that observed when studying the effects of temperature on solar cell performance. In the context of analyzing the influence of relative permittivity on device performance, a particularly pronounced impact is observed on the overall cell efficiency, mainly through a significant variation in charge transfer resistance. The resistance associated with recombination processes is also slightly affected by increasing the relative permittivity of the absorber. However, when the ratio between the absorber permittivity (*ε*_rp_) and that of the buffer layer (*ε*_rn_) approaches unity, a notable correspondence appears, this ratio highlighting a balance of the electric fields integrated at the interfaces according to the following equation.^56^16
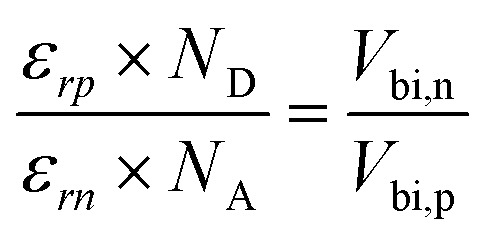


**Table 4:** Parameters obtained from modeling the effect of *ε*_r_ on the N719 dye absorber

*ε*_r_	*R*_s_ (Ω.cm^2^)	*R*_ct_ (Ω.cm^2^)	CPEp_ct_	CPET_ct_	*R*_rec_ (Ω cm^2^)	CPET_rec_ CPET_rec_
26	1.002	0.097605	0.95041	8.45 × 10^−8^	9950	1.19 × 10^−7^ 1
28	1.002	0.097283	0.95033	8.48 × 10^−8^	9949	1.22 × 10^−7^ 1
30	1.002	0.096986	0.95028	8.51 × 10^−8^	9948	1.25 × 10^−7^ 1
32	1.002	0.096711	0.9502	8.54 × 10^−8^	9947	1.28 × 10^−7^ 1
34	1.002	0.096455	0.95011	8.57 × 10^−8^	9965	1.30 × 10^−7^ 1

The Nyquist plot exhibits a profile similar to that obtained in the study of both temperature effects and permittivity on the performance of solar cells. However, a significant change is observed in the parameters associated with the charge transfer-related circuit element, particularly as the series resistance increases. This behavior is detailed in Table 5, which summarizes the parameters extracted during the analysis of the influence of series resistance.

**Table 5:** Parameters extracted from impedance modeling for *R*_s_ effect

*R*_s_ SCAPS	*R*_s_ simulation	*R*_ct_	CPET_ct_	CPEP_ct_	*R*_rec_	CPET_rec_	CPEP_rec_
1	1.002	0.096986	0.95028	8.51 × 10^−8^	9948	1.25 × 10^−7^	1
2	2.002	0.096907	9.51 × 10^−1^	8.40 × 10^−8^	9948	1.25 × 10^−7^	1
4	4.002	0.096863	0.95133	8.34 × 10^−8^	9948	1.25 × 10^−7^	1
6	6.002	0.096846	0.95148	8.32 × 10^−8^	9948	1.25 × 10^−7^	1
8	8.002	0.096836	0.95157	8.30 × 10^−8^	9948	1.25 × 10^−7^	1
10	10.0	0.096829	8.29 × 10^−8^	0.95162	9948	1.25 × 10^−7^	1

### 4.2 Electrical conductivity analysis

The electrical conductivity of a solar cell is a key parameter that reflects the ability of charge carriers (electrons and holes) to move efficiently through the different layers of the device. It strongly depends on the material properties, temperature, and frequency particularly under alternating current analysis. High conductivity facilitates efficient charge transport and reduces ohmic losses, thereby directly contributing to improved power conversion efficiency. In this study, we analyzed conductivity for the first time through the lens of the hopping conduction mechanism, providing deeper insight into charge transport phenomena under varying frequency conditions. In our case, the observed power law behavior reveals complex mechanisms such as hopping conduction and interfacial polarization effects.

**Fig. 13:**
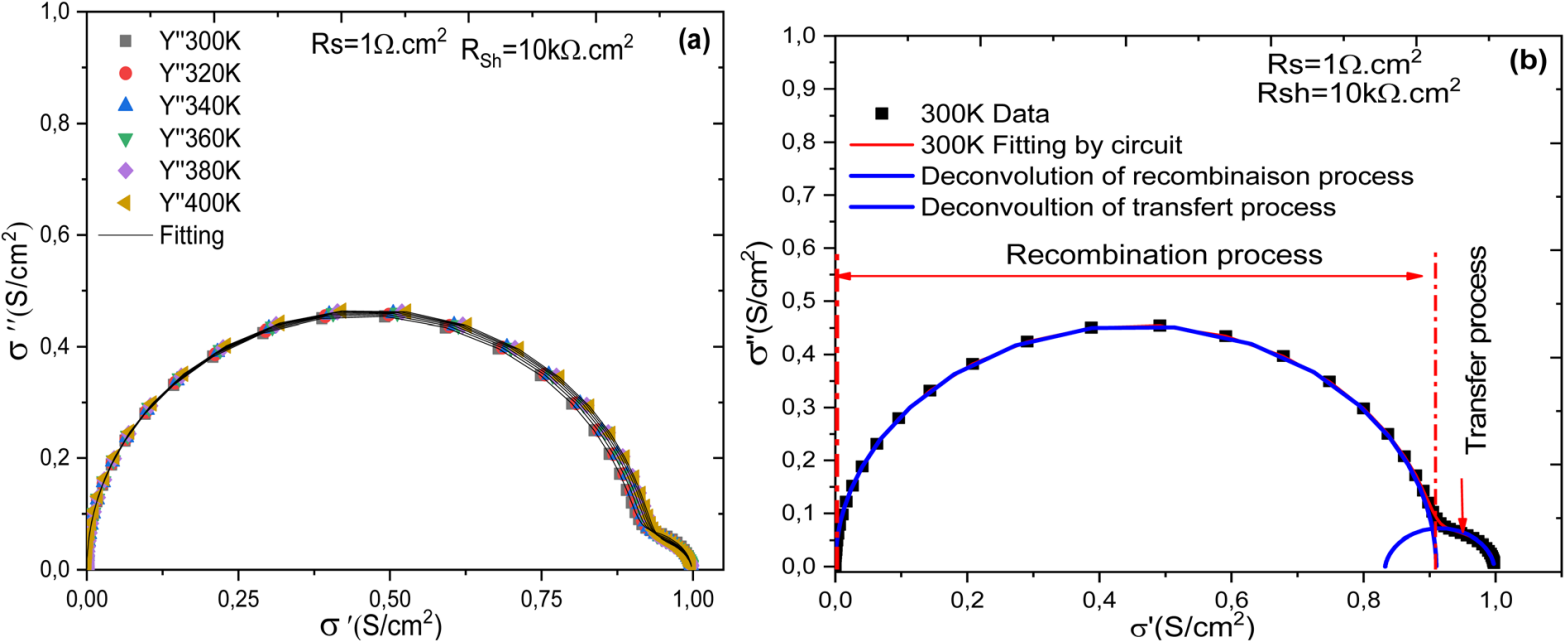
Nyquist diagram (a) and (b) deconvolution process at 300 K.

Fig. 13a illustrates the evolution of the imaginary part of the complex conductivity as a function of its real part, effectively representing the Nyquist plot of the complex conductivity for all simulated temperatures. On the other hand, Fig. 13b presents the deconvolution of the two distinct processes: charge recombination and charge transfer. This deconvolution is necessary because it is difficult to clearly distinguish the presence of two separate relaxation times using the Nyquist diagram of complex impedance shown in Fig. 9b, *vide supra* it can be observed that the conductivity of the cell undergoes two relaxation processes: one within the frequency range [1 mHz–20 MHz], followed by a second relaxation process in the frequency range [20 MHz–1 GHz]. The broadening of the dc conductivity over a wide frequency range, from 1 mHz to 100 kHz, is evident from the evolution of the conductivity as a function of frequency. The impedance measurement was analyzed and fitted with an equivalent circuit comprising two (R//CPE) connected in series. Furthermore, the ac conductivity, *σ*_ac_, was also studied as a function of frequency. It was analyzed and fitted according to a double power law, as given by the following equation:17
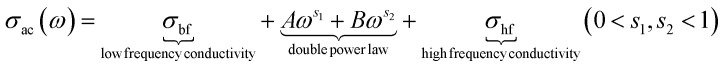


To quantify these phenomena, electrical conductivity is a valuable tool. It allows measurement of how easily charge carriers move through the material. When combined with the Nyquist plot, it enables the extraction of resistances associated with each process of charge transferandrecombination.^57^

**Fig. 14:**
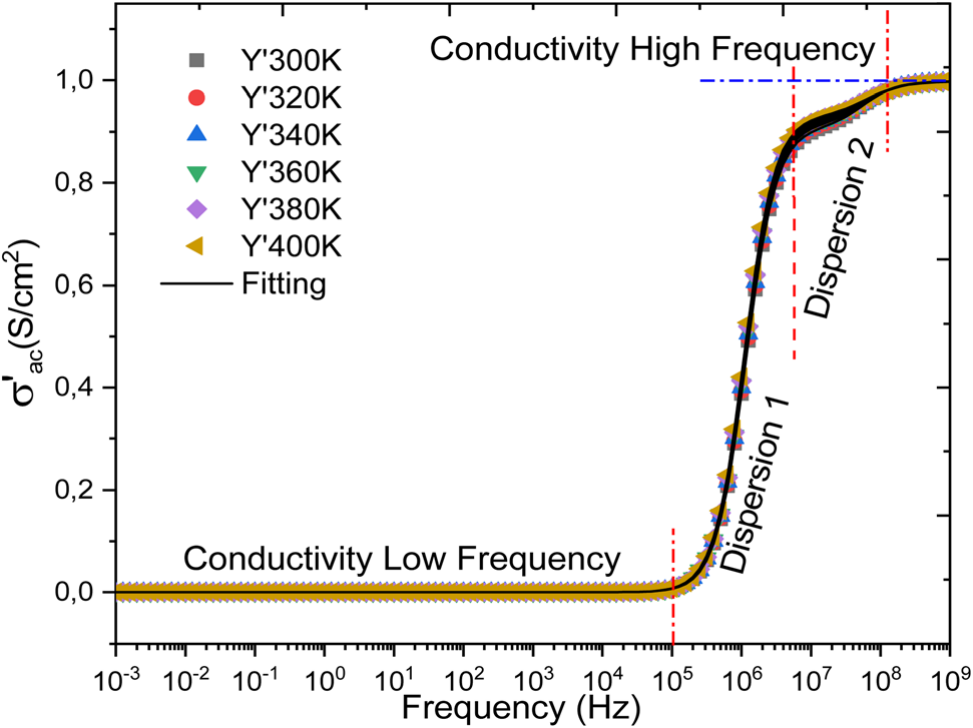
Presents the variation of the alternating current (AC) conductivity with frequency for all temperatures considered in the simulation.

Fig. 14 presents the evolution of the electrical conductivity of the cell with frequency. This graphical representation reveals that the conductivity follows Jonscher's double power law, characterized by two successive dispersion regions, labeled in the plot as dispersion 1 and dispersion 2. Three distinct conduction regimes can also be identified: region 1 corresponds to low-frequency conduction, region 2 is associated with a hopping conduction mechanism, and region 3 represents high-frequency conduction. These behaviors are described by eqn (14), which expresses the frequency dependence of the conductivity.

### 4.3 Activation energy of the charge transfer

The activation energy can be determined from the relaxation time extracted using the equivalent electrical circuit model. The relaxation time (*τ*) is typically associated with charge carrier transport or recombination processes within the solar cell.^58^ By analyzing its variation as a function of temperature, the activation energy (*E*_a_) can be derived using an Arrhenius-type relationship.^59^ This method is widely employed to reveal the nature of the dominant mechanisms that limit the dynamic response of the device, such as trap-assisted recombination or interfacial charge transfer delays.^60,61^ The equation used is as follows: 18
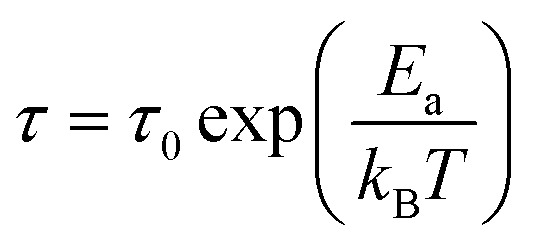


where: *τ* is the relaxation time, *τ*_0_ is the pre-exponential factor, *E*_a_ is the activation energy, *k*_B_ is Boltzmann's constant, *T* is the absolute temperature (in Kelvin).

**Fig. 15:**
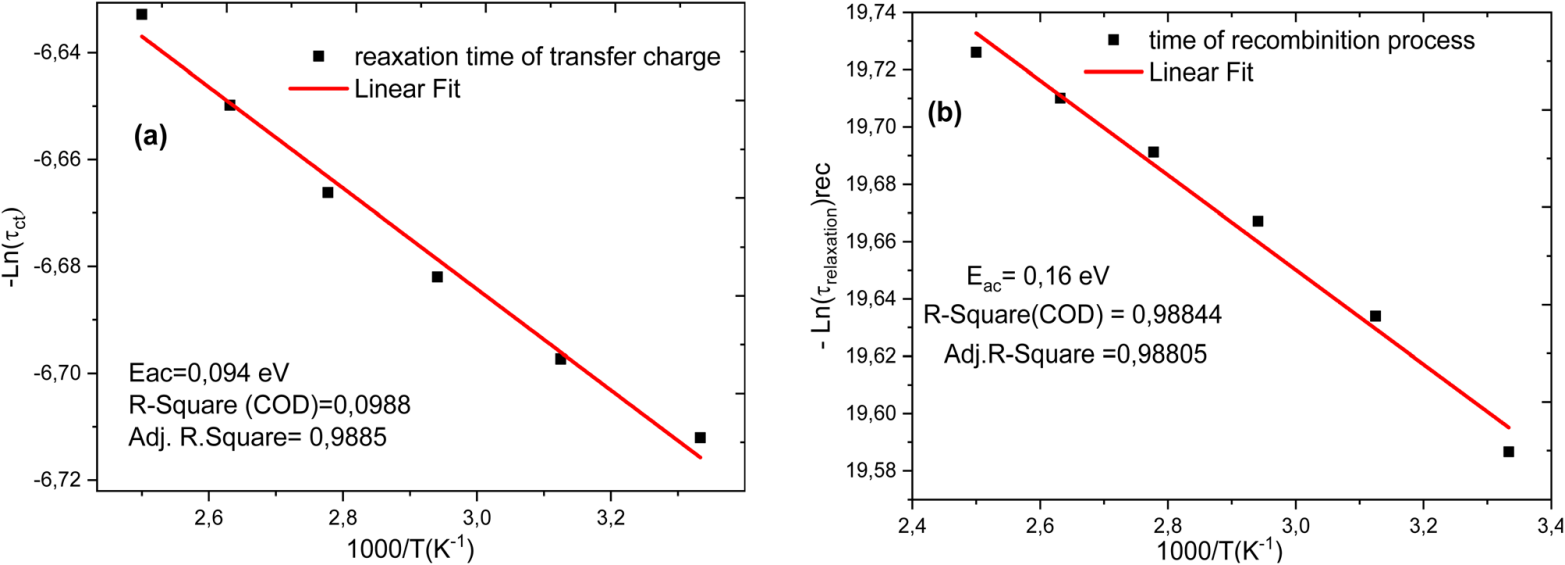
Variation of the relaxation time due to charge transfer processes (a) and recombination processes (b) as a function of 1000/*T*.

Fig. 15 presents the variation of relaxation times with the inverse of temperature. This approach has been successfully applied in various types of solar cells, including perovskite and organic devices, to analyze defect states and interfacial processes.^48,62,63^

Four scenarios can be deduced from Fig. 15:

(a) Analysis of activation energy for recombination and charge transfer processes in the Spiro-OMeTAD/N719/PC61BM/TiO_2_/FTO solar cell.

In this study, we determined the activation energies associated with the recombination and charge transfer processes in the Spiro-OMeTAD/N719/PC61BM/TiO_2_/FTO solar cell. This cell is particularly interesting due to its architecture, which includes a double ETL (electron transport layer), a factor that can significantly influence the charge transport and recombination processes.

(b) Activation energy for the recombination process.

We measured an activation energy of 0.16 eV for the recombination process in this cell. This relatively low value suggests that recombination of charge carriers occurs quite easily, even at low temperatures. Recombination in solar cells is generally a limiting factor for efficiency, as it leads to the loss of electrons and holes generated by light. A moderate activation energy, such as the one observed here, may indicate the presence of defects or imperfections in the materials, particularly at the interfaces between the different layers of the cell.

(c) Activation energy for the charge transfer process.

On the other hand, the charge transfer process, which is crucial for the efficient collection of charge carriers at the electrodes, has an activation energy of 0.09 eV. This relatively low value indicates that charge transfer occurs with a low energy barrier, which is favorable for better solar cell performance. A low activation energy for this process is often associated with high-quality interfaces between the layers, enabling more efficient injection and extraction of charge carriers. The low energy cost for this process can be partly attributed to the optimization of the ETL layers, particularly TiO_2_ and PC61BM, which facilitate electron transport.

(d) Influence of the double ETL.

It is important to note that this solar cell features a double ETL layer. TiO_2_ acts as an effective electron transport layer, while PC61BM plays a key role in improving the interface between the electrode layer and the active layer of Spiro-OMeTAD/N719. The double ETL can therefore help reduce recombination losses by providing a better path for electrons, while also increasing the efficiency of charge transfer to the electrode.^2^ The reduction in activation energy for charge transfer may also reflect this improvement in the interface due to the double ETL layer.

## 5 Comparative analysis

The performance comparison presented in Table 6 highlights the significant efficiency gains achieved in this work relative to previously reported configurations in literature. The current device outperforms earlier suggested architectures. This may be attributed to interfacial recombination and resistive losses. A more promising configuration, Pt/PEDOT: PSS/N719/PC61BM/ITO, delivers promising photovoltaic performance metrics, reflecting efficient charge extraction and reduced recombination losses at device interfaces; nevertheless, the present device depicts exceptional result primarily attributed to the use of a double ETL combination TiO_2_ and PC61BM unlike the single ETL approaches employed in the other configurations.

**Table 6:** Photovoltaic comparison between the model cell and other device architectures in literature

Configuration	Method	*V*_oc_ (V)	*J*_sc_ (mA cm^−2^)	FF (%)	PCE (%)	Ref.
FTO/PC61BM/N719/CuSCN/Au	Simul.	∼1.0	0.885	70.94	5.38	64
FTO/TiO_2_/N719 dye/CuI/Pt-FTO	Expt.	0.512	4.88	0.610	1.52	65
Pt/PEDOT:PSS/N719dye/PC61BM/ITO	Simul.	1.0691	22.38	86.64	20.73	66
FTO/TiO_2_/PC61BM/N719/Spiro-OMeTAD/C	Simul.	1.2284	25.11	86.66	26.73	This work

The double ETL structure enhances electron extraction efficiency, improves energy level alignment, and suppresses interfacial recombination, thereby boosting device electrical outcomes. The synergistic effect between TiO_2_ and PC61BM ensures rapid and selective electron transport while maintaining minimal series resistance, resulting in a high FF. The combination with Spiro-OMeTAD as the hole transport material further optimizes carrier separation and collection. These findings indicate that the proposed design not only outperforms prior configurations in simulation but also holds strong potential for experimental realization in high-performance, stable dye-sensitized solar cells.

The bilayer arrangement of TiO_2_ and PC61BM ensures better energy-level alignment across the interfaces, facilitating smoother electron transfer and minimizing recombination losses that are typically observed in single ETL systems. The synergistic interaction between TiO_2_ and PC61BM promotes rapid and selective electron transport, reduces series resistance, and enhances charge extraction efficiency.^67^ In contrast, single ETL configurations tend to exhibit greater interfacial resistance and less favorable energy matching, which limit their photovoltaic response. Furthermore, the dual ETL approach provides a balanced combination of mechanical stability and electronic functionality. TiO_2_ contributes to structural durability and optical transparency, whereas PC61BM enhances interfacial morphology and electronic contact, leading to improved overall device performance and operational stability. Thus, beyond simple optimization of a single ETL material, the TiO_2_/PC61BM strategy represents a significant advancement in ETL engineering for dye-sensitized and related thin-film solar cells.

## 6 Potential experimental challenges in the deposition of high-quality PC61BM on TiO_2_

One of the principal challenges in the fabrication of advanced dye-sensitized solar cells (DSSCs) involve incomplete pore infiltration and poor surface coverage of material layers. Mesoporous TiO_2_ films typically exhibit thicknesses of 10–15 µm and pore diameters of 10–30 nm, offering a large internal surface area.^68^ Depositing PC61BM, an organic fullerene derivative commonly processed from nonpolar solvents, into this hydrophilic nanoporous matrix is inherently difficult.^69^ The organic solution may not adequately wet the TiO_2_ surface, leading to non-uniform coating, pooling at the film surface, and uncoated regions deep within the pores. Such incomplete infiltration would compromise the intended surface passivation, enabling direct contact between the dye and unmodified TiO_2_ regions and thereby increasing recombination rates contradicting the favorable *V*_oc_ predicted by simulation. PC61BM is generally deposited from solvents such as chlorobenzene or *o*-dichlorobenzene, which are poorly suited for wetting high-temperature-sintered TiO_2_ films. These solvents may also dissolve or damage the adsorbed N719 dye layer if the deposition sequence is not carefully controlled. Furthermore, the high surface tension and low viscosity of these solvents hinder effective penetration into the nanopores, leading to non-uniform distribution and incomplete coverage. The formation of a continuous charge transport pathway is also critical for the functional performance of PC61BM as an electron conductor.^70^ Within the confined geometry of a mesoporous network, PC61BM may form discontinuous domains or isolated aggregates rather than a percolated network. This structural discontinuity would impede electron mobility, elevate series resistance, and introduce recombination centers, ultimately deteriorating device overal performance.

To address these challenges, several experimental strategies can be envisioned. One approach involves solvent and process engineering to optimize infiltration. Sequential deposition using solvents with tailored surface tension and evaporation rates potentially combined with infiltration techniques such as drop-casting, vacuum-assisted impregnation, or soaking could enable more uniform pore filling compared to conventional spin-coating.^71^ Alternatively, solvent-free deposition methods, such as thermal evaporation, may offer improved control over film uniformity and thickness, while eliminating solvent-related incompatibilities. A more integrated approach could involve the pre-formation of a TiO_2_/PC61BM composite, where PC61BM nanoparticles or precursors are blended into the TiO_2_ paste prior to film deposition. This would potentially ensure intimate contact and uniform distribution of PC61BM throughout the TiO_2_ network. However, this method would require the development of low-temperature processing techniques to avoid thermal degradation of PC61BM during sintering.

## 7 Conclusions

This study has demonstrated groundbreaking photovoltaic performance of a solid-state dye-sensitized solar cell utilizing electron transport double layers – TiO_2_ and PC61BM. The device recorded a theoretical high-power conversion efficiency of 26.73%. The optimal thickness of the absorber was found to be 2000 nm. The built-in potential (*V*_ib_) of the cell structure was considerable high at 1.0 eV, which effectively reduces the Shockley–Read–Hall (SHR) charge recombination rate leading improved cell performance. The *V*_ib_ obtained from the band alignment diagram is in agreement with the value obtained from the Mott–Schottky plot. The high *V*_ib_ observed is consistent with a remarkable *V*_oc_ of 1.2284 V. The overall optimal temperature of the cell device was observed at 300 K. The generation overwhelms the recombination by approximately an order of magnitude in the entire depth of the cell architecture. An in-depth study of the relative permittivity is essential to better understand the mechanisms of light conversion into electricity in perovskite and heterojunction solar cells. This approach is crucial for developing cells with high conversion efficiencies while minimizing series resistance. The measured activation energies for the recombination process (0.16 eV) and the charge transfer process (0.09 eV) in the Spiro-OmeTAD/N719 dye/PC61BM/TiO_2_/FTO cell provide insight into the charge carrier dynamics within this device. The low activation energy for charge transfer is promising for good cell performance, while the higher activation energy for recombination suggests that attention should be given to reducing defects and optimizing interfaces to minimize recombination losses. The presence of the double ETL layer undoubtedly plays a beneficial role in improving charge transport and reducing energy losses, thereby contributing to increased device efficiency. The predicted efficiency of 26.73% represents an aspirational upper bound for the present cell architectures and underscores the transformative role of material selection, interfacial passivation, and dielectric engineering in the next-generation photovoltaic devices. Nonetheless, realizing this concept experimentally will require overcoming substantial fabrication challenges, particularly those associated with the uniform deposition of PC61BM within mesoporous TiO_2_ frameworks and reconciling the idealized modeling assumptions with material and interfacial realities. Future experimental efforts should, therefore, focus on process optimization, alternative deposition routes, and comprehensive interfacial characterization to bridge the gap between simulation and practice.

## Author contributions

GGN: conceptualization, writing original draft, method development, data analysis & editing, AE: method development, supervision, numerical analysis, writing & editing, ELM: method development, supervision & editing, JKK: conceptualization, method development, supervision, simulation & editing. All authors have read and approved the manuscript.

## Conflicts of interest

The authors have no competing interests.

## Data Availability

The data associated with the findings of this study are available from the corresponding author upon reasonable request.
